# A Review of Converging Technologies in eHealth Pertaining to Artificial Intelligence

**DOI:** 10.3390/ijerph191811413

**Published:** 2022-09-10

**Authors:** Iuliu Alexandru Pap, Stefan Oniga

**Affiliations:** 1 Department of Electric, Electronic and Computer Engineering, Technical University of Cluj-Napoca, North University Center of Baia Mare, 430083 Baia Mare, Romania; 2 Department of IT Systems and Networks, Faculty of Informatics, University of Debrecen, 4032 Debrecen, Hungary

**Keywords:** eHealth, mHealth, telehealth, telemedicine, remote patient monitoring, Internet of Things, brain–computer interface, artificial intelligence, machine learning, deep learning

## Abstract

Over the last couple of years, in the context of the COVID-19 pandemic, many healthcare issues have been exacerbated, highlighting the paramount need to provide both reliable and affordable health services to remote locations by using the latest technologies such as video conferencing, data management, the secure transfer of patient information, and efficient data analysis tools such as machine learning algorithms. In the constant struggle to offer healthcare to everyone, many modern technologies find applicability in eHealth, mHealth, telehealth or telemedicine. Through this paper, we attempt to render an overview of what different technologies are used in certain healthcare applications, ranging from remote patient monitoring in the field of cardio-oncology to analyzing EEG signals through machine learning for the prediction of seizures, focusing on the role of artificial intelligence in eHealth.

## 1. Introduction

The COVID-19 pandemic has pushed healthcare systems to their limits, while highlighting the critical demand for reliable, efficient, and secure remote healthcare services that can be provided even throughout lockdowns or other disease-spread control measures.

In this paper, we try to offer an overview of the plethora of technologies and different approaches used in the latest eHealth implementations. Many eHealth applications use embedded systems and employ the use of artificial intelligence for the analysis of biomedical data.

With the continued improvement of different technologies, from authentication methods to artificial intelligence data analysis, many are adopted by emerging eHealth applications, IoT implementations and software frameworks. While most of these technologies are not related to health, their combined advantages lead to a greater development pace in multiple eHealth projects and bridge the gap between researchers.

Because eHealth is a broad term used to describe all applications that have appeared in recent years, within this review, we will classify the discussed research into categories better defined by subdomain terms such as mHealth, telehealth and telemedicine. MHealth stands for mobile health and is better described as eHealth applications requiring mobile devices, namely, smartphones, tablets or wearable devices. Telemedicine can be associated with any clinical method that delivers care at a distance through technology. Telehealth is another frequently used eHealth subdomain referring to everything telemedicine stands for, plus other non-clinical services, such as promoting, preventing, training and educating practitioners.

The role of telehealth and some additional insights into the development of healthcare during the COVID-19 pandemic, especially in association with artificial intelligence (AI), are detailed in [1].

The primary benefit of AI algorithms is that they are implemented in a way that mimics certain aspects of human thinking, and they can handle various structured or unstructured types of input data when aiming to solve a specific problem. There are multiple subsets of AI, one of them being machine learning (ML). ML is a method used to train a model from datasets without explicitly programming it. The model can then be used to replicate the learnt behavior on new data. There are several approaches to ML, some of them being:

- Supervised learning: learning starts with a set of example inputs and their correct outputs.
- Unsupervised learning: only input data are provided and the algorithm groups or clusters the data, giving it a structure.
- Semi-supervised learning: some of the data are missing training labels.
- Reinforcement learning: focused on learning through trial and error.

Deep learning (DL) is a subset of ML that employs multiple layers to extract features from raw input data.

AI is one of the key technologies responsible for the evolution of eHealth, bringing multiple advantages to the field of healthcare, such as the following:

- Improvements in diagnosis accuracy, e.g., detecting heart failure [2].
- Risk prediction, e.g., predicting antibiotic resistance through machine learning [3], cardiovascular disease prediction from AI-based models [4], and using deep learning to predict cardiac indices [5].
- Clinical applications, e.g., nutrition assessment [6].
- Healthcare process optimization, e.g., the complexity and the potential of integrating AI into healthcare processes [7].
- Patient flow, e.g., enhancing patient flow for mental health [8,9].
- Precision medicine, e.g., detecting sleep apnea through deep learning [10].
- Automate detection, e.g., automated acute myocardial infarction detection using ECGs from smartwatches [11].
- Improved quality of care, e.g., using AI to improve chronic disease care for type 2 diabetes mellitus patients [12].
- Reduced healthcare costs, e.g., predicting health and population well-being [13].
- Discovering adverse effects of medication, e.g., machine learning models discovering adverse drug effects on the gut microbiome [14].

## 2. Overview of Healthcare Applications

The four main categories in which we can organize eHealth applications are: eHealth (implementations that do not belong in any of the following subdomains), mHealth, telehealth and telemedicine. Sometimes more narrow classifications can be made, such as:

- Remote patient monitoring (RPM)—refers to patient monitoring and the transmission of medical data such as blood pressure, heart rate, heart rhythm, oxygen saturation, glucose levels, weight, etc. Clinicians can often monitor this data in real time.
- Interactive patient care—offers live interactive communication between patients and healthcare service providers via video, phone or other secure channels.
- Store and forward—consists of recording and transmitting multimedia data such as image, sound or video.

Remote patient monitoring, interactive patient care and store and forward are all included in the telemedicine category.

### 2.1. eHealth

During the COVID-19 pandemic, healthcare service provisioning became the primary focus of many researchers experimenting with innovative technologies such as blockchain and artificial intelligence [15].

An eHealth application [16] within the COUCH project (under the European Union’s Horizon 2020 R&D program) was developed for and tested on older adults with some health problems. The application used text-based dialogue to interact with the participants. This study attempted to assess how an agent-based eHealth platform would be used in a real-world environment, and if it had any health effects on the participants.

An ever-evolving procedure that has seen major improvements regarding its research accessibility in recent years is electroencephalography (EEG). While recording EEG data has become cheaper and easier because of equipment availability and performance, it is still challenging to record consistently and successfully analyze the acquired data.

Portable, low-power IoT devices such as the Texas Instruments MSP432 can be used in conjunction with an efficient seizure-predicting algorithm with great results, even if the number of EEG electrodes is reduced [17].

Because of the nature of the EEG-recorded data, many researchers opt to replace the costly feature extraction methods with deep learning models that use recurrent neural networks (RNNs) such as long short-term memory (LSTM). In [18], using LSTM for sequence classification, the proposed system is able to identify persons that have previously suffered concussions by recording EEG data while the person is concentrating on a task.

In Table 1, we can observe the spectrum of different technologies used in eHealth applications over the past couple of years.

By combining filter banks and Riemannian tangent space (FBRTS), a fusion method for feature extraction in motor imagery brain–computer interfaces (MI-BCIs) is proposed in [19], with good classification accuracy in both datasets it was tested in, and great applicability in eHealth by interpreting EEG signals.

A wearable Internet of Things (WIoT) hybrid edge-cloud online stress-monitoring solution [20] can achieve a much higher accuracy when offloading data to the cloud models compared to the edge ones.

The importance of data confidentiality has led researchers to work on creating privacy-preserving seamless authentication frameworks, optimizing authentication processes by reducing the number of computations and offering users anonymity through dynamic identities in smart Internet of Things (IoT) implementations [21], attempting to enhance the security of healthcare systems and to secure the transmission of data between intended parties.

A more specialized branch of IoT is HIoT (healthcare IoT), or IoMT (Internet of Medical Things), where the objective is to build medical applications that can monitor a patient’s health, prevent health issues, or predict disease.

### 2.2. mHealth

In many fields, predicting a health crisis has long been limited to the classic method of collecting useful information from patients. These methods imply direct contact with the patient for a significant amount of time, either in person or by video calls.

Newer and smarter technologies allow clinicians and researchers to collect data about the patient’s everyday activities through noninvasive methods by continuously gathering sensor data from devices such as smartphones, smartwatches and other personal gadgets.

By analyzing this information, the emergence of specific patterns can be observed and then correlated to different health events.

One such use of passively collected data to determine biomarkers for mood states can be seen in [29], where emotional state prediction models were tested for clinical outpatients that had previously been diagnosed with mental disorders, owned smartphones with Android or iOS operating systems, and had them connected to Wi-Fi networks at least a couple of times per week.

Cardiac monitoring wearable implementations can offer a wide range of applications, often targeting similar types of biomedical data.

Standard electrocardiogram (ECG) measurements offer glimpses of electrical signals over a short period of time and any arrhythmia that occurs outside that time interval is overlooked. A solution to this problem is a continuous ECG monitoring system that is not prone to overlook certain episodes.

There are multiple types of cardiac monitoring, as stated in [30], where different aspects are discussed:

1. Advantages and disadvantages of wearable solutions for arrhythmia monitoring (outpatient telemetry);
2. Wearables can also deliver therapy (electric shocks in life-threatening situations);
3. they can improve the quality of life (early discharge, less outpatient visits, reassurances);
4. Ultra-portable electrocardiogram patches (Zio Patch by iRhythm Technologies, San Francisco, CA, USA) that can last up to 14 days without recharging and can, in some scenarios, provide better diagnostic yield than Holter monitoring;
5. Sleep apnea screening can be undertaken with wrist-worn reflective photoplethysmography, which has a promising correlation to standard polysomnography;
6. Artificial intelligence algorithms can be used in conjunction with the data collected from wearables, automatically detecting multiple conditions, without the need for manual interpretation.

In [31], we are presented with an attempt to create a model with the exclusive purpose of predicting psychotic relapses in conditions such as schizophrenia spectrum disorders. The data required for this work were obtained from a system composed of an Android application and a cloud-based platform. An encoder–decoder neural network was used to provide anomaly detection based on the collected passive sensing data.

Similarly to the previous table, in Table 2 we are presented with mHealth implementations, their results and the technologies used.

### 2.3. Telehealth

Local eHealth systems can provide deep learning capabilities for processing biomedical signals along with collecting the necessary raw data.

Remote patient monitoring systems and telemedicine implementations often face difficulty remotely acquiring the necessary sensor data from the patient.

Screening patients in person offers the possibility of using advanced sensors and specialized equipment. When this is unattainable, one solution is to extract new kinds of data from the existing connection the system has with the patient to be able to identify useful risk markers for conditions not yet recognized.

Telehealth implementations could screen patients for different conditions by recording the video feed and analyzing it for potential markers.

One such implementation, a sensor-less deep learning image-processing frailty meter [36] records 20 s elbow movement videos (flexion and extension) through the camera of a tablet and then the frailty phenotype and frailty index are calculated, thus screening the patient for physical frailty and having the means to triage patients with chronic obstructive pulmonary disease (COPD).

Remote healthcare services for the treatment, management and remote monitoring of Parkinson’s disease patients should be built, taking in consideration the fact that neurological examinations carried out in the hospital can sometimes offer less information about the patient’s symptoms than prolonged observations, because some impairments can occur at specific times during the day, hence the need to use wearable devices that act as automated systems [37].

IoT systems that allow in-home health monitoring have been adopted in recent years thanks to their many advantages achieved by leveraging technologies such as dedicated communication protocols (LoraWan, Sigfox, NB-IoT), the 3GPP standard, smarter medical devices and sensors (blood pressure, pulse, glucose meter, temperature, blood oxygen saturation, ECG, accelerometers, etc.), devices supporting wireless technologies (Zigbee, Wi-Fi, Bluetooth, BLE, NFC, RFID, etc.) and cloud computing services for analyzing the previously collected data [38].

Accessible development boards such as the Raspberry Pi make excellent testing devices because of their low cost and versatility, translating to new possibilities for eHealth and telehealth systems. There are numerous commercially available ways of connecting a great number of sensors to the Raspberry Pi. To list the most useful features, these boards offer configurable general-purpose input–output pins (GPIOs), audio and video connectors, RJ-45 Ethernet or wireless connectivity and USB ports.

In our previous work with EEG signals [39,40], we used the same e-Health sensor platform as in [41], where arrhythmia detection is possible through ML algorithms using a Raspberry Pi, an Arduino and the e-Health Sensor Platform v1.0. The e-Health sensor platform allows an Arduino or the Raspberry Pi through an adaptor shield to collect data from blood pressure monitors, pulse oximeters, galvanic skin response, airflow, and temperature sensors.

Previously described works and others can be found in Table 3, where we can discover telehealth applications, their purpose, and the technologies they employ.

### 2.4. Telemedicine

The 2019 SARS-CoV-2 pandemic impacted most aspects of daily life, but the healthcare sector especially was extremely affected. During this challenging period, the field of telemedicine has seen a great expansion both in its use and in its functionalities.

Arguably, the most significant benefit of telehealth applications is the fact that patients are not required to travel back and forth. If the consultation can be carried out remotely, commuting to the clinic or hospital is at least impractical and possibly even pointless.

Especially during the pandemic, patient waiting times became significantly more unpleasant due to mask wearing and social distancing policies, which meant that waiting rooms could not be filled entirely and many patients had to wait outdoors, regardless of the weather conditions. In situations where patients had to travel great distances even before an initial consultation, the added strain on their bodies could have posed a health risk.

In large regions such as Western Australia, teleophthalmology solutions were offered to rural patients while different artificial intelligence approaches were experimented with to identify age-related macular degeneration, diabetic retinopathy and glaucoma [44].

In a review that spanned over 3 years of PubMed articles related to teleophthalmology, including the first 2 years of the COVID-19 pandemic, the importance of telemedicine was assessed with regard to the reduction in manpower, limiting direct patient contact, more efficient medical information storage, and real-time diagnosis [45].

Telemedicine applications have begun assisting ambulance crews because the emergency services deploy rapid response teams consisting of nurses and volunteers, but not doctors. By using a Raspberry Pi and a commercially available webcam, the wearable solution of medical tele-tutoring REC-VISIO 118 can provide a stabilized video of the situation, mainly for first aid interventions, that can be accessed through a web interface in real-time through a 4G connection, leading to a faster pre-diagnosis for time-dependent conditions such as heart attacks or strokes [46].

The need for artificial intelligence is emphasized in [47], pointing to the ever-expanding volume of sensor data that must be interpreted in order to find any useful information, while satisfying the common needs of the stakeholder groups responsible for the actual development and implementation.

Considering that the second cause of death in patients that overcome cancer is cardiovascular disease, the COVID-19 pandemic has raised concerns regarding cardiovascular toxicities and the way clinicians react to them, taking into account innovations during and after the pandemic, as follows [48]:

1. Digital health technologies such as mobile health (mHealth) and wearables help patients to monitor their own health and reduce the number of unneeded hospital visits, detect abnormal heart rates (Apple Watch), and collect and analyze health data, caring for patients while keeping them safe during the pandemic.
2. In the field of telemedicine, many freely accessible cloud-based solutions have become compliant with laws that protect patient information, making cardio-oncology able to benefit from telehealth.
3. Social media-assisted telehealth, through its various means of information propagation, has an important impact on prevention and innovation, especially in cardio-oncology.
4. Artificial intelligence is without a doubt one of the foundation elements of many eHealth implementations, be it mHealth, wearables, remote patient monitoring or other applications. By connecting artificial intelligence algorithms with social media platforms, these systems can not only predict some conditions, but even help locate or get in contact with the patient.

Table 4 brings together relevant telemedicine applications, along with a description of their results and the technologies they used to implement them.

## 3. Challenges of eHealth and AI Applications in Healthcare

As we can appreciate from the previously mentioned papers, AI is starting to play an instrumental role in healthcare, no matter what branch we look at, but its integration in healthcare is not as straightforward.

For electronic healthcare to successfully adopt solutions offered by AI, ML or DL, the challenges that eHealth is currently facing must be overcome. Analyzing these challenges, we explore the salient ones in the following subsections.

### 3.1. Adoption

To increase the adoption rate of AI in healthcare, we need to examine how AI is perceived and what requirements need to be met, as in [53].

AI adoption in the public healthcare system can be facilitated by applications such as [54], where family health education is introduced to artificial intelligence, proposing an AI-based family health education public service.

AI plays an important role in analyzing large amounts of data in precision medicine, but in public health, with much smaller amounts of information per patient, its use is not as justifiable. As the trends tend to merge precision medicine and public health into precision public health [55], AI adoption is one of the best solutions to analyzing big data.

With [56], we are presented with a multiphase plan to speed up the adoption of artificial intelligence in healthcare by:

1. Identifying the difficulties in utilizing the power of AI in care delivery;
2. Creating education plans for multiple interventions;
3. Determining curriculum issues;
4. Increasing awareness, introducing certificate-based interventions for healthcare providers and for leaders, and providing coaching and innovation hubs;
5. Performing evaluation studies;
6. Encouraging sharing best practices and the creation of new knowledge.

### 3.2. Security and Privacy of Health Information

One major concern in utilizing any technology that transmits or manipulates healthcare records is the security and privacy of such information. In [57], healthcare records are protected from malicious-intent users through a combination of artificial intelligence-based agents and smart contracts generated through the use of blockchain technology, making sensitive health records distributed and immutable. The mixture of eHealth and blockchain for the purpose of protecting health data seems to provide a well-acknowledged solution, as we see in [23] and in combination with AI in [15].

Some attempts at securing healthcare environments use encryption and the cloud, in addition to AI, for the management of the blood bank supply chain with reduced human intervention [58].

### 3.3. Usability of AI in eHealth

Platforms such as the CARTIER-IA [59] can provide a solution to the ease-of-use issue concerning non-specialized users by implementing design decisions focused on creating an accessible interface to organize, preview or manipulate medical data or imagery and, most importantly, to make use of AI algorithms regarding the available medical information without needing specialized AI training.

### 3.4. Accessibility and Affordability

While artificial intelligence can provide revolutionary insights in ways human-based data analysis cannot, for many low-income countries, the cost and availability of such technologies can be a real obstacle. Studies such as [60] provide a counterargument, showing the role and the level of efficiency machine learning algorithms can have in predicting the mortality of children under five years old in low-to-middle income countries.

### 3.5. Ethical and Social

Artificial intelligence has raised many concerns, especially when human-based roles are to be handed over to a machine capable of making decisions regarding the wellbeing of human beings, where errors could potentially lead to death. Such concerns, as well as accountability, bias, responsibility, trust, and the risks of the dehumanization of care, are analyzed in studies such as [61,62].

## 4. Discussion

Artificial intelligence is present in numerous implementations that need to analyze large amounts of data and, since sensors are more freely available, more eHealth applications are likely to be compelled to use AI algorithms.

The rhythm of innovation in eHealth has reached new highs, which is to be expected if we consider the coronavirus pandemic, but also the pace at which wearables, virtual reality, augmented reality, and the technologies highlighted in this review are being developed, leading to an evolved healthcare system.

From [63], we can extract hypothetical future perspectives on artificial intelligence and eHealth, along with trends such as patient involvement for evaluating the quality of care and satisfaction, offering care for patients with multiple morbidities, shifting to value-based healthcare, and using precision medicine to treat patients individually.

As AI breaches new frontiers into different medical fields, it is prone to face divergent perspectives about its usefulness. Various legal and ethical issues may arise, leading to new challenges and concerns, such as AI and the future of psychiatry [64,65], whether or not AI can replace tasks that mental health practitioners perform, and how ethical aspects have received little attention.

While software-controlled machines become more prevalent, unsettling claims arise about robots replacing doctors, highlighting the importance of artificial empathy development [66].

**Figure 1:**
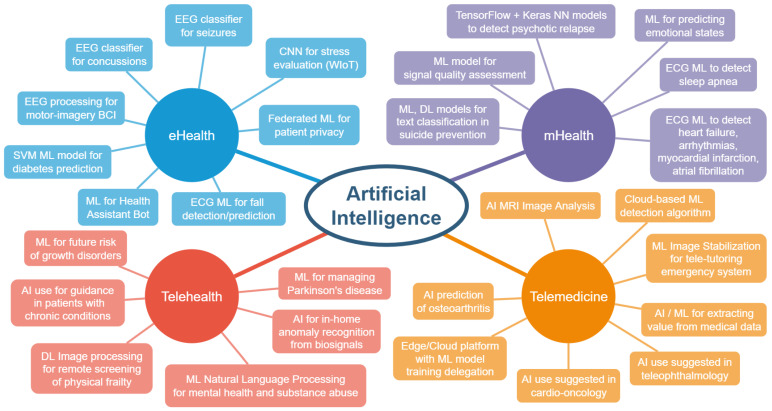
Artificial intelligence utilization in eHealth.

To provide a better overview, we constructed Figure 1 from the classifications presented in this review, showing the existing and proposed applications of AI by category.

Future research is welcomed and encouraged in fields such as prognosis and health management (PHM) [67], digital pathology (DP) [68], disease diagnostics [69], regenerative surgery [70], AI electrocardiogram [71], remote health monitoring (RHM) [72], and AI documentation assistance for consultations in primary care [73].

From our perspective, there are many technologies that are on a convergent course with healthcare, bringing additional benefits to each field they employ. We think the following future directions complement what previously mentioned works have highlighted:

- Social networking tends to play an important role in our daily lives, even if we try to minimize it as much as possible. Teenagers and young adults are highly interconnected through this communication medium, meaning that they can more easily be influenced by wrong advice received from persons pretending to be medical professionals. To address this issue, we believe that some eHealth solutions should incorporate social components into their implementations, providing a trusted source of information. From [74], we are presented with the connection of social media and vaccine hesitancy, pointing out the importance of the presence of actual health experts in social media discussions.
- Surveillance camera presence is continuously increasing in many homes, marketplaces, public and industrial places. Still, a limited number of security cameras offer certain human movement detection. We strongly believe that this perpetually alert watchdog could safeguard our lives as well. COVID-19 has proven that cameras can be crucial in detecting face masks [75], aiding both healthcare and law enforcement agencies. One of the low-cost approaches to fall detection, according to the review in [76], is the use of camera-based devices. A future direction we see developing at least in testing, after taking into consideration privacy concerns and anonymizing the data, consists of surveillance nodes sending information to processing servers where fall-detection events could be confirmed and acted on to the extent of notifying healthcare services. This method could provide solutions for public or workplace accidents, where the only witness is the surveillance camera. The processing power could be provided by the healthcare service or an organization, unrelated to the detection node’s sector.
- Cryptocurrency is present in most modern fintech mobile applications, offering various benefits and capabilities that have yet to find applications in eHealth. In [77], a dedicated cryptocurrency coin called Wholesome Coin is introduced with the hope of making people healthier and reducing medical costs. With the development of WIoT, many new ways of motivating users to live healthier lives are emerging.
- The gamification of health could be another direction that provides improved health services with less traumatic experiences for children. The Xploro platform [78] has reduced the procedural anxiety of children attending hospitals.

## 5. Conclusions

Our primary focus in this paper was to gather a diverse collection of works from the past couple of years that provide the most realistic insights into what technologies are being adopted in eHealth, regarding the use of artificial intelligence. Through this review, by presenting the plethora of ways AI can be used in eHealth applications, we also try to underline different healthcare sectors affected by the COVID-19 pandemic that can benefit from integrating AI technologies into their systems to increase performance and handle health crises more efficiently.

## Figures and Tables

**Table 1:** eHealth applications.

Authors	Work Description	Results	Technologies Employed
[15]	Decentralized, patient-centric healthcare system framework	Interoperability between healthcare platform stakeholders; patients own their data; challenges include the volume of raw clinical data, privacy and security.	Artificial intelligence, blockchain
[16]	Virtual coaching system for older adults	Increasing elders’ engagement with a conversational agent-based eHealth platform to provide modern healthcare services to less tech-savvy patients; study limitations include selection bias, lack of personalized content and testing remotely because of COVID-19 restrictions.	Functional demonstrator of eHealth application COUCH
[17]	Epileptic seizure prediction embedded system using EEG	Good accuracy with reduced number of electrodes; low power consumption; running on IoT devices; measurements of energy consumption and execution time for processing EEG data segments; EEG data from ambulatory monitoring system with 16 electrodes, 400 Hz sampling rate in 10 min clips.	Texas Instruments MSP432 low-power device, EEG, IoT
[18]	System used to identify persons that suffered concussions through EEG analysis	High accuracy of classifier (92.86%); data consisted of 46 recordings with 63 channels, 4–6 min of EEG data; data analysis was challenging.	Artificial intelligence/deep learning model based on long short-term memory (LSTM)
[19]	Feature extraction for motor imagery brain–computer interface	New method with good classification accuracy evaluated on two datasets (used 22 EEG channels from the 9 participants included in BCI competition IV dataset 2a and 2b).	Brain–computer interface, novel filters, EEG
[20]	Stress monitoring system for everyday use	Hierarchical edge-cloud obtains lower response time by 77.89% and energy consumption by 78.56%; models in the cloud; high computational effort and missing data proved challenging.	Artificial intelligence, CNN, IoT, Wearable IoT
[21]	IoT smart eHealth system authentication that preserves privacy	Improved transmission rate resulting in more active users; verified by simulations with NS-3 tool.	Cryptosystem, MAC verification
[22]	Body area network-based wearable fall detection system	Efficient system that can analyze substantial amounts of data in real-time; data recorded from an ECG sensor with 3 channels and 4 accelerometer nodes.	Body area network (BAN), acceleration, ECG sensors
[23]	A blockchain-based system for detecting medical document changes and notifying patients	The system does not upload medical records but notifies the patients if the documents have been changed.	Blockchain, mobile app development
[24]	Fuzzy-based trust management for preventing Sybil attacks on Internet of Medical Things systems	Proposed model recognizes compromised Sybil nodes and declares them malicious; Sybil attacks are difficult to detect.	Internet of Medical Things (IoMT), trust management
[25]	Personal health assistant using the Italian language	System with conversational agent that monitors treatments and biological values and is able to suggest doctors; the generated probability dataset can be used for 217 diseases.	Artificial intelligence/machine learning, chatbot, Telegram-based
[26]	IoT-based eHealth surveillance system designed for pandemics	Using geographic routing algorithms to monitor persons for health conditions, social distancing, and mask-wearing status.	GPS, Node-RED, Influx, Grafana
[27]	A system for analyzing and predicting diabetes mellitus	Tested prediction results on real hospital data collected from 500 patients presenting risk factors of developing diabetes mellitus.	Artificial intelligence/machine learning, K-nearest neighbor
[28]	LI-Care system for health monitoring	Cost-efficient monitoring system with GUI offering powerful signal acquisition and processing; data rates of the sensors used in this work are between 120 B/s and 10 KB/s.	LabView, IoT, National Instruments myRIO-1900

**Table 2:** mHealth applications.

Authors	Work Description	Results	Technologies Employed
[29]	Emotional state prediction through machine learning techniques	Personalized models; data collected through eB2 MindCare; 943 users selected; limitations of the study include missing observations and sporadically reported emotional states information.	Artificial intelligence/machine learning, smartphones
[30]	Cardiac monitoring system based on smart wearables	Review of real-world use of arrhythmia and other cardiovascular devices	Artificial intelligence, remote patient monitoring, wearables, ECG
[31]	Predicting psychotic relapse in patients with schizophrenia spectrum disorders (SSDs)	Better prediction of anomalies in patients with SSDs; 20,137 days of data collected through CrossCheck study; anticipated challenges during deployment.	Artificial intelligence/machine learning, smartphones, Android application CrossCheck
[32]	Signal quality assessment algorithm to classify the signal quality of ECG and respiratory	Signal quality classification with good accuracy; challenges: signal quality misjudgment, most SQAs were not conducted by daily life use of wearable devices, best methods are supervised ML models.	Wearable device (SensEco)
[33]	Adoption of voice interface technology for patients with heart failure	Higher remote engagement between patients and providers for better heart failure prevention; data from 47 patients; challenges: engagement and ease of use.	Technology based on Amazon’s Alexa voice assistant (Alexa+) with Echo Dot devices; Avatar tablet application (Avatar);
[34]	Review on mobile health use in atrial fibrillation	Expert claims ECG, PPG (photoplethysmography) and MCG (mechanocardiography) use in medicine can reduce morbidity.	Wearables for PPG and ECG; handheld devices for MCG, PPG and ECG; remote monitoring
[35]	Boamente, a suicidal prediction mobile application	Identifies suicidal ideations from texts originating from a virtual keyboard; dataset built using Twitter API and labeling tweets by psychologists; dataset sharing restricted by Twitter’s policy.	Artificial intelligence/deep learning, neural language processing, digital phenotyping

**Table 3:** Telehealth applications.

Authors	Work Description	Results	Technologies Employed
[36]	Application capable of remotely screening patients for physical frailty	Using a technology as accessible as a tablet camera, this remote screening solution extracts kinetic features and calculates a frailty index; results were compared with other solutions; dataset built from 11 patients.	Artificial intelligence/deep learning, remote patient monitoring, tablet video recording, video processing
[37]	A review of Parkinson’s disease management systems at home	Remote management and automated assessment of Parkinson’s disease wearable systems	Wearables, accelerometers, gyroscopes, mobile apps, web technologies, SSL, SSH, VPN, TLS
[38]	A review of IoT in-home health monitoring systems	Presented works offer a wide view over IoT implementations for in-home health monitoring systems	IoT, ambient assisted living, LoraWan, Sigfox, NB-IoT, 3GPP, RESTAPI, ECG and other medical sensors, Zigbee, Bluetooth, BLE, NFC, RFID, etc.
[41]	Secured telehealth system for IoT capable of biosignals diagnosis	The system can handle multiple types of sensors through an Arduino board; a Raspberry Pi 3 model B+ is used for processing the data; uses 4 Physiobank databases: MIT-BIH Arrhythmia, MIT-BIH Normal Sinus Rhythm, BIDMC Congestive Heart Failure and MIT-BIH AF.	Artificial intelligence/machine learning, C# app, EEG, Xbee modules, e-Health sensor platform, Raspberry Pi
[42]	Mental health and substance abuse telehealth	Researchers analyzed tweets and concluded there were 4 times more tweets relating to mental health and substance abuse during the pandemic compared to before; data cleaning was challenging because some originated from organizations; selected 10,689 tweets.	Artificial intelligence / machine learning, natural language processing, social media, Twitter
[43]	Review relating to telehealth in pediatric endocrine disorders	Precision medicine; growth hormone therapy; diabetes patient care.	Artificial intelligence, IoT, specialized devices to deliver injections that use a web platform

**Table 4:** Telemedicine applications.

Authors	Work Description	Results	Technologies Employed
[44]	Telemedicine solution for eyecare in remote Western Australia	Shorter patient waiting time for first consultation, reduction in costs, availability in remote regions, detecting multiple conditions remotely; faced logistical and geographical challenges;	Artificial intelligence, video conferencing, store and forward methods
[45]	A review about the evolving role of teleophthalmology in a COVID-19 pandemic	Describes the use of teleophthalmology, expanding [44].	Artificial intelligence, video conferencing
[46]	A telemedicine system used to offer emergency assistance through a wearable helmet	The REC-VISIO 118 is used daily in the 118 Emergency Service of Pistoia on COVID-19 suspects; video data are transmitted via the 4G network.	Artificial intelligence/machine learning, WebRTC, webcam video recording and transmission, image stabilization, IoT (system based on Raspberry Pi 3), 4G communication
[47]	A review discussing cardiovascular problems offering telemedicine solutions	Various solutions are discussed, presenting advantages, challenges and solutions for digital health tools.	Artificial intelligence/machine learning, various wearables
[48]	Review regarding cardio-oncology patient care during COVID-19	Presents possible ways of using big data, social media and AI to provide care for cancer survivors, because cardiovascular diseases are the second cause of death among this group.	Artificial intelligence/machine learning, social media, big data
[49]	Review about rheumatology challenges in telemedicine	Advantages and shortcomings of telemedicine in rheumatology, some studies even showing that telemedicine did not reduce the face-to-face consultations.	Artificial intelligence/machine learning, mobile applications, wearables, remote patient monitoring
[50]	Telemedicine health analysis system based on IoT	The researchers propose a cloud IoT architecture to improve the connection between health IoT and people, providing detailed analysis of different layers; challenges associated with big data management and processing.	Artificial intelligence, IoT, quality of service framework, quality of experience, cloud
[51]	Research the effect of telemedicine on gestational diabetic patients	A reminder system and telephone access were used to improve healthcare efficiency, while having little to no impact on the blood glucose levels; difficulties with computer access and low-income families; dataset of 80 patients equally split in intervention and randomizing dot control groups.	Short message service (SMS), interactive voice response (IVR)
[52]	On-demand orchestration of services for health emergency predictions	The CURATE system supports scaling to better respond to rising simultaneous prediction requests received from the edge; work presents benefits of continuous IoT health monitoring via 5G service orchestration two-tiered platform (edge-cloud); system used time series data from two ECG channels; training phase consists of 10 epochs of 200 steps;	Network Functions Virtualization Management and Orchestration (NFV MANO), 5G Public–Private Partnership Infrastructure Association, Cloud/Edge, Tensorflow/Keras (Python)

## Data Availability

Not applicable.
